# Black tea protects against hypertension-associated endothelial dysfunction through alleviation of endoplasmic reticulum stress

**DOI:** 10.1038/srep10340

**Published:** 2015-05-15

**Authors:** Wai San Cheang, Ching Yuen Ngai, Ye Yen Tam, Xiao Yu Tian, Wing Tak Wong, Yang Zhang, Chi Wai Lau, Zhen Yu Chen, Zhao-Xiang Bian, Yu Huang, Fung Ping Leung

**Affiliations:** 1 Institute of Vascular Medicine and Li Ka Shing Institute of Health Sciences, Chinese University of Hong Kong, Hong Kong, China; 2 Department of Cardiovascular Sciences, Methodist Research Institute, Houston, TX, USA; 3 School of Life Sciences, Chinese University of Hong Kong, Hong Kong, China; 4 Clinical Division, School of Chinese Medicine, Hong Kong Baptist University, Hong Kong, China

## Abstract

Hypertensive patients have been found to be associated with elevated levels of homocysteine, known as hyperhomocysteinemia. Homocysteine (Hcy) can induce endoplasmic reticulum (ER) stress in endothelial cells. This study aims to investigate whether black tea (BT) protects against hypertension-associated endothelial dysfunction through alleviation of ER stress. Rat aortae and cultured rat aortic endothelial cells were treated with Hcy, BT extract, and theaflavin-3,3’-digallate (TF3). Male Sprague Dawley rats were infused with angiotensin II (Ang II) to induce hypertension and orally administrated with BT extract at 15 mg/kg/day for 2 weeks. Hcy impaired endothelium-dependent relaxations of rat aortae and led to ER stress in endothelial cells, which were ameliorated by co-incubation of BT extract and TF3. The blood pressure of Ang II-infused rats and plasma Hcy level were normalized by BT consumption. Impaired endothelium-dependent relaxations in renal arteries, carotid arteries and aortae, and flow-mediated dilatations in third-order mesenteric resistance arteries were improved. Elevations of ER stress markers and ROS level, plus down-regulation of Hcy metabolic enzymes in aortae from Ang II-infused rats were prevented by BT treatment. Our data reveal the novel cardiovascular benefits of BT in ameliorating vascular dysfunctions, providing insight into developing BT into beneficial dietary supplements in hypertensive patients.

Clinical investigation suggests a positive association between systolic hypertension and hyperhomocysteinemia^1^. Homocysteine (Hcy) is a sulfur-containing amino acid that is produced during the metabolic demethylation of methionine. Studies show that an elevated blood Hcy level is an independent risk factor for coronary heart disease, stroke, and peripheral vascular disease^2^. It is also reported that Hcy induces endoplasmic reticulum (ER) stress in human vascular endothelial cells^3^,^4^. Disruption of protein folding, trafficking and degradation results in the accumulation of unfolded proteins in the ER and this condition is defined as ER stress. ER stress plays a significant role in vascular dysfunction during hypertension^5^ and suppression of ER stress improves endothelium-dependent contraction in aortae of spontaneously hypertensive rats^6^. To date, the possible role of ER stress in hypertension-induced hyperhomocysteinemia and subsequent endothelial dysfunction remain largely elusive.

There has been a mounting interest in understanding cardiovascular and metabolic benefits of polyphenolic flavonoids in tea, which can be used as a supplement among patients. Compelling evidence in human and murine models also suggest various cardio-protective benefits of consuming tea or tea polyphenols under pathological conditions, including hypertension, atherosclerosis, diabetics, hypercholesterolemia, and obesity; and are attributed to antioxidative, anti-thrombogenic, anti-inflammatory, hypotensive and hypocholesterolemic properties of tea polyphenols^7^,^8^,^9^,^10^. However, a recent systemic review and meta-analysis of randomized controlled trials shows that green tea catechin supplementation does not reduce plasma C-reactive protein, a pro-inflammatory marker^11^.

Green tea epicatechins relax rat arteries probably through stimulating nitric oxide release^12^,^13^. We previously discussed the cardiovascular benefits of tea polyphenols in four aspects including (i) vasorelaxant effect; (ii) protective effect against endothelial dysfunction; (iii) antioxidant effect and (iv) hypolipidemic effect^14^. The present study delineates that black tea protects against endothelial dysfunction in angiotensin II (Ang II)-induced hypertensive rats through the inhibition of ER stress and oxidative stress in the vascular wall.

## Results

### Black tea extract and theaflavin-3,3’-digallate (TF3) reduced Hcy-induced endothelial dysfunction in rat aortae through alleviation of ER stress

**Figure 1:**
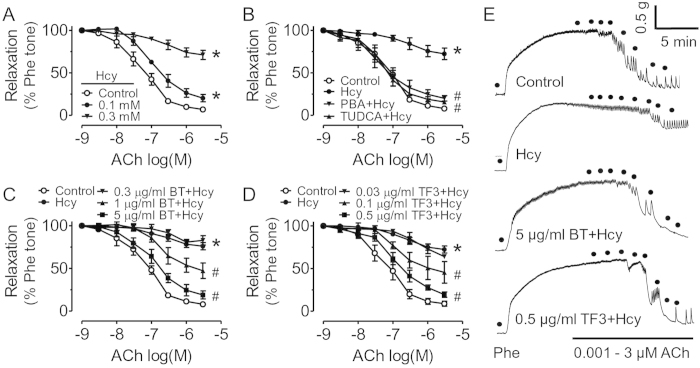
Black tea extract (BT) or theaflavin-3,3’-digallate (TF3) improve acetylcholine (ACh)-induced endothelium-dependent relaxations (EDRs) in rat aortae that were impaired by homocysteine (Hcy). (**A**) Hcy *ex vivo* incubation for 45 min reduced EDRs in a concentration-dependent manner in rat aortae contracted by phenylephrine (Phe). (**B**) Pretreatment for 30 min with endoplasmic reticulum (ER) stress alleviator 4-phenyl butyric acid (PBA, 10 μM) and tauroursodeoxycholic acid (TUDCA, 20 μM), (**C**) BT or (**D)** TF3 significantly improved EDRs in Hcy-treated rat aortae. (**E)** Representative traces of EDRs in different groups. Results are means ± SEM of 4-5 separate experiments. *p < 0.05 vs Control. #p < 0.05 vs Hcy.

**Figure 2:**
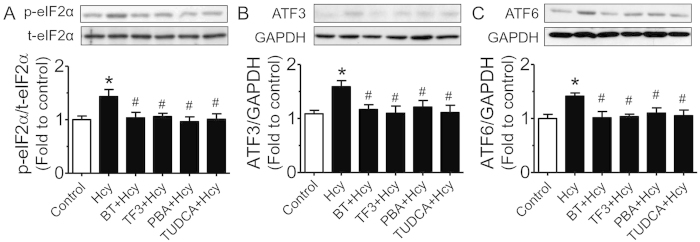
BT and TF3 suppress Hcy-induced ER stress in rat aortic endothelial cells (RAECs). ER stress markers such as (**A)** phosphorylated eIF2α at Ser^52^ (p-eIF2α) compared to total eIF2α (t-eIF2α; 36 kDa), (**B**) ATF3 (21 kDa) and (**C**) cleaved ATF6 (50 kDa) compared to housekeeping GAPDH (37 kDa) were elevated by 45 min-incubation with Hcy in RAECs which was inhibited by 30 min-pretreatment of BT (5 μg/ml), TF3 (0.5 μg/ml), PBA (10 μM) and TUDCA (20 μM). Results are means  ±  SEM of 6 separate experiments. *p < 0.05 vs Control, #p < 0.05 vs Hcy.

To examine whether black tea extract (BT) and TF3 (as one of main theaflavins in black tea) restore endothelial function through alleviation of ER stress, acetylcholine (ACh)-induced endothelium-dependent relaxations (EDRs) were examined. The EDRs of rat aortae were impaired by treatment with Hcy (0.01 – 0.3 mM) for 45 min (Fig. 1A). In addition, pretreatment of ER stress alleviators 4-phenyl butyric acid (PBA) and tauroursodeoxycholic acid (TUDCA) improved EDRs that were impaired by Hcy in rat aortae (Fig. 1B). Pre-incubation of black tea (0.3 – 5 μg/ml) and TF3 (0.03 - 0.5 μg/mL) for 30 min improved EDRs that were impaired by Hcy (Fig. 1C,D). Representative traces in Fig. 1E show that EDRs were impaired in Hcy-treated rat aortas and they were restored after treatment of BT extract and TF3. Furthermore, BT extract, TF3, PBA and TUDCA all reduced phospho-elF2α (Ser^52^), ATF3 and cleaved ATF6 protein levels (ER stress markers). Hcy-treated primary cultured rat aortic endothelial cells (RAECs) (Fig. 2).

### Black tea extract improved endothelial function in Ang II-infused hypertensive rats through alleviation of ER stress

**Figure 3:**
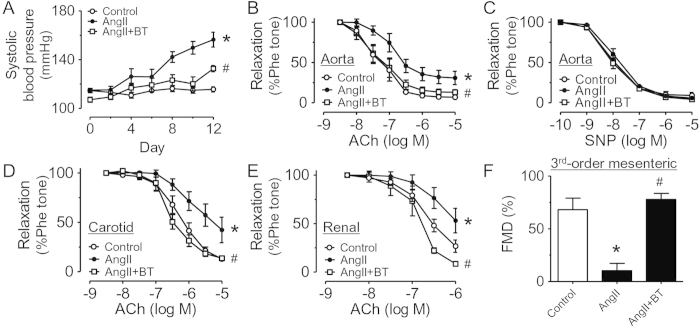
BT treatment enhances vascular function in Ang II-infused hypertensive rats. (**A**) Changes in systolic blood pressure in Ang II-induced hypertension (Ang II) with BT treatment (Ang II  +  BT). Concentration–relaxation curves for (**B**) ACh in aortae, (**C**) sodium nitroprusside (SNP) in aortae, (**D**) ACh in carotid arteries, and (**E**) ACh in renal arteries from control, (**F**) Flow-induced dilatation in third-order of mesenteric arteries. Results are means ± SEM of 5-7 rats. *p < 0.05 vs Control, #p < 0.05 vs Ang II.

**Figure 4:**
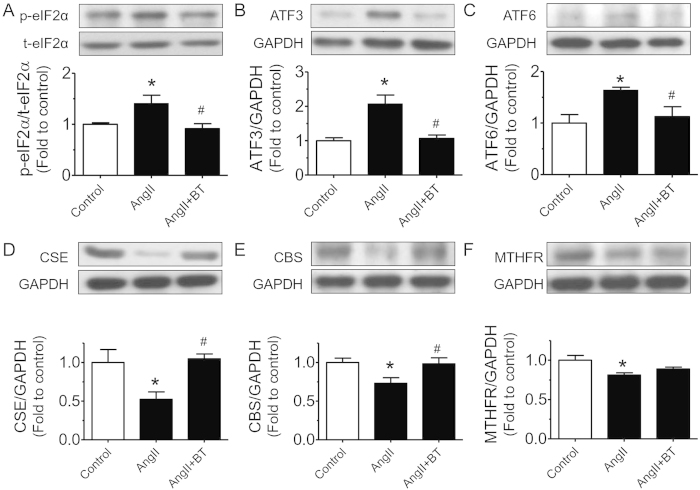
BT treatment alleviates ER stress in aortae from Ang II-infused hypertensive rats. Effect of chronic BT treatment on (**A**) phosphorylated eIF2α at Ser^52^ (p-eIF2α) compared to total eIF2α (t-eIF2α; 36 kDa), (**B**) ATF3 (21 kDa) and (**C**) cleaved ATF6 (50 kDa) compared to housekeeping GAPDH (37 kDa) in aortic rings from Ang II-treated rats. BT treatment restored protein expression of (**D**) CBS and (**E**) CSE, and (**F**) slightly elevated MTHFR level compared to housekeeping GAPDH (37 kDa) in aortae from Ang II = induced hypertension. Results are means ± SEM of 5-7 rats. *p < 0.05 vs Control, #p < 0.05 vs Ang II. The full-length blots/gels are presented in Supplementary Figure 2.

Ang II induced high systolic blood pressure reaching ~156 mmHg within 2 weeks of osmotic infusion, which was attenuated by treatment with BT extract (Fig. 3A). In Ang II-infused rats, treatment with BT extract prevented the augmentation of plasma Hcy whereas body weight and heart weight were not affected (Table 1). Moreover, BT extract improved EDRs in aortae, carotid and renal arteries of Ang II-infused rats (Fig. 3B,D,E) without affecting the sodium nitroprusside-induced endothelium-independent relaxations (Fig. 3C). Moreover, the impaired flow-mediated dilatations of third-order rat mesenteric arteries were also restored by treatment with BT extract (Fig. 3F). The expressions of ER stress markers such as phosphorylated elF2α at Ser^52^, ATF3 and cleaved ATF6 were greater in aortae from Ang II-infused rats compared to those from normotensive rats. BT extract reversed the up-regulated levels of ER stress markers *in vivo* (Fig. 4).

### Black tea restored protein levels of Hcy metabolic enzymes in Ang II-induced hypertension

Since hypertension is associated with high levels of Hcy, we determined whether this was attributed to Hcy metabolism. Western blot analysis revealed that the protein levels of Hcy metabolic enzymes, cystathionine-β-synthase (CBS), cystathionine gamma-lyase (CSE) and methylenetetrahydrofolate reductase (MTHFR), were decreased significantly in Ang II-treated rats (Fig. 4 D,E,F). Following BT extract treatment, protein levels of CBS and CSE increased significantly and MTHFR expression slightly in the Ang II + BT group.

### Black tea attenuated ROS production *in vivo* and *in vitro*

**Figure 5:**
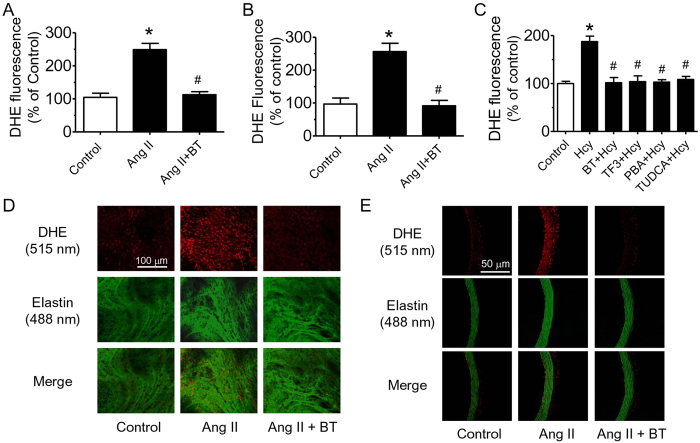
BT treatment reduced hypertension-associated or Hcy-stimulated reactive oxygen species (ROS) over-generation in rat aortae and endothelial cells. ROS production estimated by dihydroethidium (DHE) staining in (**A)** *en face* endothelium and (**B**) cross-sections from control, Ang II, and Ang II + BT rats. (**C**) Decrease of ROS by 30 min-pretreatment of BT (5 μg/ml), TF3 (0.5 μg/ml), PBA (10 μM) or TUDCA (20 μM), followed by 45 min- treatment of Hcy in RAECs. Representative images of DHE staining in (**D)** *en face* endothelium and (**E**) cross-sections. Red, DHE fluorescence (excitation: 515 nm) in the nucleus; green, autofluorescence of elastin underneath the endothelium (excitation, 488 nm). Results are mean ± SEM of 4-5 separate experiments. *p < 0.05 vs Control, #p < 0.05 vs Ang II or Hcy.

A reduction in ROS levels was observed following BT extract treatment in aortae (both *en face* endothelium and cross-sectional area) from Ang II-induced hypertensive rats (Fig. 5A,B,D,E). Dihydroethidium (DHE) staining showed that 30 min-pretreatment with black tea extract and TF3 as well as ER stress alleviators, PBA and TUDCA, reversed Hcy-stimulated ROS production in RAECs (Fig. 5C).

## Discussion

The present study provides novel evidence showing the protective benefits of black tea extract against endothelial dysfunction in hypertensive rats through alleviation of ER stress. We observed impaired vasodilatation, up-regulated levels of plasma Hcy, reduced Hcy metabolic enzymes, and increased ER stress markers and ROS generation in aortae from chronic Ang II-infused rats, which were reversed by chronic administration of black tea extract *in vivo*. The major novel results include (i) black tea extract and its major theaflavins TF3 *ex vivo* reverse Hcy-induced endothelial dysfunction and Hcy-elevated ER stress in rat aortae; (ii) black tea extract treatment normalizes plasma Hcy levels in Ang II-infused rats, and mitigates high blood pressure; (iii) chronic black tea extract treatment reverses the attenuated endothelium-dependent relaxations in aortae, carotid and renal arteries as well as flow-mediated dilatations (FMD) in third-order mesenteric resistance arteries, accompanied by inhibition of ER stress and up-regulation of Hcy metabolic enzymes (CBS and CSE) in the Ang II-infused rats; and (iv) black tea extract and TF3 normalized the elevated ROS generation in RAECs treated with Hcy and in the aortae of Ang II-infused rats.

Hyperhomocysteinemia has been reported to contribute to the development of hypertension as a result of endothelial dysfunction^15^. Endothelial dysfunction was indeed pronounced in patients with hypertension with risk factors of atherosclerosis and coronary heart disease^16^. Possible mechanisms involve increased oxidative injury to the endothelium, proliferation of vascular smooth muscle cells, and elevation of vascular adhesion protein-1 (VAP-1) and alteration of arterial structural components such as collagen, elastin, and proteoglycans^15^,^17^.

In addition, high levels of Hcy reduce bioavailability of nitric oxide and impair relaxations^18^. Higher Hcy concentrations are associated with systemic arterial stiffness and high blood pressure response to stress in hypertensive patients^19^ and healthy subjects^20^. Hcy is known to trigger ER stress in vascular endothelial cells, activating ER stress markers including GPR78, ATF4, ATF3, IRE1, and JNK^21^,^22^. Our *ex vivo* experiments were in agreement with previous studies supporting that Hcy led to ER stress and endothelial dysfunction in rat aortae. Such impairment was reversed by treatment with black tea extract and TF3. Evidences have implied that consumption of black tea protects cardiovascular function not only by scavenging ROS, but also by promoting eNOS activity through PI3/Akt pathway^23^,^24^. The present study is probably the first to show that black tea polyphenols can relieve ER stress and subsequently enhance endothelial function. ER stress has been demonstrated to be closely associated with pathogenesis of obesity, insulin resistance and type 2 diabetes^25^. More importantly, ER stress induced by high glucose^26^, free fatty acids^27^, and oxidized and glycated LDL cholesterol^28^, causes endothelial dysfunction and atherosclerosis. We have recently reported that ER stress contributes to vascular dysfunction in obese and diabetic mice; and that alleviation of ER stress restores vascular function^29^. The vascular benefits of black tea were confirmed *in vivo* in Ang II-induced hypertension which is associated with hyperhomocysteinemia and ER stress through normalizing blood pressure and restoring vascular function in various arteries.

In consistence with previous study^30^, Ang II-induced hypertension increased plasma Hcy level and diminished protein levels of transsulfuration enzymes (CBS and CSE) and remethylation enzyme (MTHFR) in Hcy metabolism. Of note, chronic consumption of black tea extract lowered plasma Hcy level and restored the protein levels of CBS and CSE in aortae in Ang II-infused rats. These results indicate that black tea polyphenol decreased Hcy levels in hypertension by promoting Hcy metabolism.

Hypertension occurs in approximately 30% of Western populations and is known to be a major cause of stroke, heart failure, and myocardial infarction. Nonetheless, the molecular etiology of hypertension remains poorly understood. ER stress is suggested to be a new paradigm for Ang II–induced hypertension^31^. ER stress is mediated by three ER membrane–associated proteins that engage complex downstream signaling pathways, including cleavage of ATF6, activation of eIF2α/ATF3 pathway, and splicing of X-box binding protein 1^32^. Some of these pathways were exaggerated in Ang II-infused rats and were suppressed by black tea extract. This study provides new insight into the molecular mechanisms that drive hypertension and suggests a potential target for future therapy. Our *ex vivo* findings demonstrate that treatments of black tea extract and TF3 reduced ER stress, inhibiting the expressions of phosphorylated eIF2α, ATF3 and cleaved ATF6. The attenuation of ER stress contributed to the improved endothelial function as ER stress alleviators PBA and TUDCA also possessed similar effects. Likewise, our *in vivo* results support that black tea extract treatment restores the impaired EDRs by reducing ER stress.

ER stress and oxidative stress are closely linked events^33^. ROS are produced during normal ER metabolism. Accumulating evidence reveals that under stress conditions such as ER stress, ROS production is increased via enzymes of the NADPH oxidase (Nox) family, especially via the Nox2 and Nox4 isoforms, which are involved in the regulation of blood pressure^34^. Oxidative stress has been implicated in many diseases associated with protein misfolding, such as diabetes, and atherosclerosis^35^. Compelling evidence supports the notion that antioxidants and diet modification can alleviate oxidative stress in these disease states^36^. The present study suggests that chronic treatment with black tea extract is highly effective to reduce oxidative stress in Ang II-infused rats. The improvement in endothelial function of hypertensive rats was attributed to the decrease of both ER stress and oxidative stress. Given that PBA and TUDCA reduced ROS generation *in vitro*, the inhibitory effect of black tea extract and TF3 on oxidative stress is at least partially mediated through alleviation of ER stress.

Recent meta-analyses show that chronic consumption of tea can lower blood pressure and even reduce cardiovascular mortality^37^,^38^,^39^,^40^, nevertheless, the human intervention studies only link the benefits to improved bioavailability of nitric oxide (NO). ER stress can also affect NO level as reported in our previous study: ER stress inducer tunicamycin reduced NO production in endothelial cells^29^. The present study provides a novel mechanistic insight that chronic black tea consumption reduces ER stress and oxidative stress, and likely with positive effect on NO bioavailability, to protect endothelial function and lower blood pressure.

In summary, angiotensin II infusion increases plasma Hcy level which leads to ER stress and oxidative stress, and subsequently endothelial dysfunction. Chronic administration of black tea extract confers protection against ER stress and endothelial dysfunction in arteries from hypertensive rats. The findings herein are consistent with *ex vivo* results in rat aortae treated with Hcy. Black tea administration reduced blood pressure and plasma Hcy. The present data also strengthen the prospective of the potential use of black tea polyphenols as therapeutic agents or health supplement for patients with hypertension and hyperhomocysteinemia. The present study highlights the idea that ER stress provides new avenues for investigation and may lead to new therapeutic approaches against hypertension.

## Materials and Methods

### Animal treatment

Male Sprague Dawley rats (250-260 g) were purchased from Chinese University of Hong Kong Laboratory Animal Service Centre. Animals were assigned into the following groups: (1) Vehicle (saline), (2) Ang II, (3) Ang II + Black tea extract (BT). The *in vivo* effect of Ang II was evaluated by systemic infusion of Ang II (1000 ng/kg/min, dissolved in saline) into rats subcutaneously by osmotic minipumps (Alza Corp., Palo Alto, CA, USA), at the dose of 50 ng/kg/min. Black tea extract was given at 15 mg/kg/day/ by oral gavage starting from Day 1 after Ang II pump insertion and continued for 14 days. Animals were sacrificed at Day 15. The animals were housed under conditions of controlled temperature (23 ± 2 °C) and humidity (60%) with 12 h light/dark cycles. All the experimental procedures were approved by the Animal Ethnics Committee of the Chinese University of Hong Kong. All experiments were carried out in accordance with the approved guidelines.

### Systolic blood pressure by tail-cuff method

The systolic blood pressure was measured using the CODA tail-cuff blood pressure system (Kent Scientific, Torrington, USA). Arterial blood pressure measurements were carried out at the same time of the day so as to avoid the influence of the circadian cycle, and the value for systolic blood pressure was obtained.

### Artery preparation and functional study by organ bath and wire myograph

Rats were sacrificed by CO_2_ inhalation. The thoracic aortae, carotid arteries and renal arteries were dissected and cleaned of adhering connective tissue in ice-cold and oxygenated Krebs-Henseleit solution containing (mM): 119 NaCl, 4.7 KCl, 2.5 CaCl_2_, 1 MgCl_2_, 25 NaHCO_3_, 1.2 KH_2_PO_4_, and 11 D-glucose. Each aorta was cut into several ring segments (~3 mm in length) for parallel studies, and each experiment was performed on rings obtained from different rats. The aortic ring was suspended between two stainless steel hooks in a 10-ml organ bath filled with Krebs solution. Likewise, carotid and renal arteries were cut into ring segments (~2 mm in length) and were suspended between two stainless steel wires in Multi Myograph System (Danish Myo Technology, Denmark) with 5-ml Krebs solution. Bathing solution was continuously bubbled with 95% O_2_ and 5% CO_2_ and maintained at 37 ^o^C (pH of 7.3-7.5). All rings were initially stretched to an optimal resting tension (aortae: 2.5 g; carotid arteries: 10 mN; renal arteries: 3 mN) and equilibrated at 37 ^o^C for 60 min before the start of experiments^41^,^42^. Rings were contracted with 60 mM KCl and rinsed in Krebs solution. Each ring was then contracted by phenylephrine (Phe, 1 μM). Once a sustained tension was reached, either ACh (3 nM-10 μM) or SNP (1 nM -10 μM) was added cumulatively to evoke endothelium-dependent or endothelium-independent relaxations, respectively.

In one set of experiments, rat aortae with intact endothelium were exposed to Hcy at three concentrations (0.01 - 3 μM) for 45 min before the determination of ACh-induced EDRs. In another set of experiments, rat aortae were incubated with PBA (10 μM), TUDCA (20 μM), BT (0.3-5 μM), or TF3 (0.03-0.5 μM) for 30 min and further incubated with Hcy for 45 min, followed by examination of vascular reactivity.

### Flow-mediated dilatation (FMD) in pressure myograph

The third-order rat mesenteric artery (external diameter: 280-350 μm) was dissected free of surrounding adipose tissue and was cannulated between two glass cannulas in a chamber filled with 10 mL of oxygenated Krebs solution^42^,^43^. The intraluminal pressure and vessel diameter were monitored by a light-inverted microscope (Zeiss Axiovert 40 microscope, model 11 P) with video camera, and the Myo-View software (Danish Myo Technology). Under no-flow condition, the artery segment was subjected to stepwise increment of 20 mmHg in intraluminal pressure from 20 to 80 mmHg at 5-minute intervals at 37 °C and 3 µM phenylephrine was added to induce vasoconstriction after the vessel’s diameter stabilized. FMD was triggered by pressure change that equals ~15 dynes/cm^2^ shear stress. FMD was calculated as the percentage of phenylephrine-induced constriction by the following equation: FMD = (Df - Dphe/Di - Dphe) ×100% where D represents the external diameter of vessels; Df is the maximum vessel diameter after flow; Dphe is the diameter after phenylephrine constriction and before flow; Di is the initial diameter without any treatment.

### Primary culture of RAECs

Rat thoracic artery was dissected free connective tissue and cut open longitudinally in sterile phosphate buffered saline (PBS). The stripe was digested with 0.2% collagenase (Type IA, Sigma-Aldrich, St. Louis, MO, USA) in PBS (w/v) for 15 min at 37 ^o^C. The detached endothelial cells were collected by centrifugation at 1500 rpm for 10 min and resuspended in RPMI 1640 medium containing 10% fetal bovine serum plus 100 U/ml penicillin and 100 μg/ml streptomycin (Invitrogen, Carlsbad, CA, USA). After 1-hr incubation at 37 ^o^C, the medium was refreshed to remove unattached cells and endothelial cells were cultured in an incubator with 5% CO_2_ at 37 ^o^C till 80-90% confluence.

### Western blot analysis

Aortae or RAECs were homogenized in ice-cold RIPA lysis buffer and the lysates were centrifuged at 20,000 g for 20 min at 4 ^o^C to collect supernatants. The protein concentration was determined using the Lowry method (Bio-rad, Hercules, CA, USA). Protein samples (20 μg) were separated with 10% SDS–PAGE and transferred to a nitrocellulose immobilon-P polyvinylidene difluoride membrane (Millipore, Billerica, MA, USA) using wet transfer (Bio-Rad) at 4 ^o^C. Non-specific binding sites were blocked by 5% non-fat milk or 1% BSA in 0.05% Tween-20 phosphate-buffered saline, and then incubated at 4 °C overnight with primary antibodies against eIF-2α (1:1000, Cell signaling Technology, Beverly, MA, USA), phosphorylated eIF-2α at Ser^52^ (1:1000, Invitrogen), ATF3 (1:1000, Santa Cruz Biotechnology, Santa Cruz, CA, USA), ATF6 (1:500, Abcam, Cambridge, UK), CSE (1:500, Abcam, Cambridge, UK), CBS (1:500, 1:500, Abcam, Cambridge, UK), MTHFR (1:500, Abcam, Cambridge, UK), GAPDH (1:10000, Ambion, Austin, TX, USA). The blots were incubated with appropriate secondary antibodies at a 1:3000 dilution for 1 h at room temperature, and then washed 3 times for 20 min in PBST. The membranes were finally developed with an enhanced chemiluminescence detection system (ECL reagents, Amersham Pharmacia, Pittsburgh, PA, USA), and finally exposed to X-ray films. Equal protein loading was verified with use of GAPDH as housekeeping protein. Densitometry was performed with a FluoChem documentation system (Alpha Innotech Corp., San Leandro, CA, USA) and analyzed with QuantityOne software (Bio-Rad).

### ROS determination

ROS measurement in *en face* endothelium and cross-sectional area of rat aortae was performed as described^43^. Some aortic rings were freshly prepared for *en face* staining of ROS while some aortic rings were embedded in OCT compound (Tissue-Tek) for cutting into sections of 10-μm thickness on cryostat (Shandon, Pittsburgh, PA, USA). Fresh aortae, frozen sections of aortic rings, or cultured RAECs were incubated for 20 min with 5 μM DHE (Molecular Probes, Eugene, OR, USA)-containing PBS at 37 ^o^C. Fluorescence was observed by Fluoview FV1000 laser scanning confocal system (Olympus, Tokyo, Japan; 515-nm excitation; 585-nm long pass filter). DHE fluorescence intensity was analyzed by Fluoview FV10-ASW1.5 software. The summarized data represent the fold change in fluorescence intensity relative to that in control rat aortae.

### Plasma Hcy measurement

Blood was collected by cardiac puncture and immediately centrifuged to get the plasma which was then stored at –80 °C until analysis. The plasma Hcy levels were determined by the kit according to the manufacturer instructions (Catalog #80454, Crystal Chem, Inc., IL, USA).

### Drugs and chemicals

ACh, Phe, SNP, TUDCA and PBA were purchased from Sigma-Aldrich. Black tea extract (product code: QP-Black tea extract; source: *Camellia sinensis O. Ktze, Batch #: TJtf130311*) and TF3 (QP-1023, Batch #: TJ13031223) were purchased from Quality Phytochemicals LLC (East Brunswick, NJ, USA). The certificate of analysis of the black tea is presented in the supplementary materials. The black tea extract was assayed by routine HPLC methods (Supplementary Figure 1 and Table 1) and the analysis showed the specification of theaflavins as 70% and the analyzed active ingredients of the black tea extract are theaflavins, theaflavin-3-gallate, theaflavin-3’-gallate, and theaflavin-3,3’-digallate.

### Data Analysis

Results represent means ± SEM of n separate experiments. Relaxations were expressed as percentage reduction in phenylephrine-induced contraction. Data were analyzed by one-way ANOVA followed by Bonferroni post hoc tests whenever appropriate (GraphPad Software, San Diego, USA). P < 0.05 indicates statistical difference between groups.

## Author Contributions

W.S.C., C.Y.N., X.Y.T., Y.Y.T., C.W.L., W.T.W. and Y.Z. performed the experiments and analyzed data. W.S.C. prepared Figs. 1 and 2. C.Y.N. prepared Figs. 3,4 and Table 1. F.P.L. and Z.Y. prepared Fig. 5. F.P.L. and W.S.C. wrote the manuscript. F.P.L., Z.X.B., Z.Y.C. and Y.H. designed the study and reviewed the manuscript.

## Additional Information

**How to cite this article**: Cheang, W.S. *et al.* Black tea protects against hypertension-associated endothelial dysfunction through alleviation of endoplasmic reticulum stress. *Sci. Rep.* **5**, 10340; doi: 10.1038/srep10340 (2015).

## Supplementary Material

Supporting Information

## Figures and Tables

**Table 1:** Body weight, heart weight, the ratio of heart weight to body weight, and plasma homocysteine (Hcy) level for control, Ang II, and Ang II + BT rats.

**Parameters**	**Control**	**Ang II**	**Ang II + BT**
**Body weight (g)**	311.3 ± 8.22	307.5 ± 13.06	305.0 ± 8.07
**Heart weight (g)**	1.26 ± 0.04	1.30 ± 0.04	1.28 ± 0.04
**Heart/body weight (%)**	0.41 ± 0.01	0.42 ± 0.01	0.42 ± 0.01
**Plasma Hcy (μM)**	28.18 ± 3.41	53.36 ± 7.40^*^	27.45 ± 3.80#

Data are presented as mean ± SEM of 5 rats, *p < 0.05 vs Control, #p < 0.05 vs Ang II.
