# Supplementary material for: Black tea protects against hypertension-associated endothelial dysfunction through alleviation of endoplasmic reticulum stress
Source: Sci Rep. 2015 May 15;5:10340. doi: 10.1038/srep10340 (PMC4432571; doi:10.1038/srep10340)
Supplement: Supporting Information [file srep10340-s1.pdf]

Title: Black tea protects against hypertension-associated endothelial dysfunction through alleviation of endoplasmic reticulum stress

Authors: Wai San Cheang, Ching Yuen Ngai, Ye Yen Tam, Xiao Yu Tian, Wing Tak Wong, Yang Zhang, Chi Wai Lau, Zhenyu Chen, Zhao-Xiang Bian, Yu Huang, Fung Ping Leung

Supplementary material

The manufacturer “Quality Phytochemical LLC” has provided the certificate of analysis of the black tea extract.

Quality Phytochemicals LLC

Certificate of Analysis

Product Name	Theaflavins
Product Code	QP-Black Tea Extract
Synonyms:	TF
Source:	*Camellia sinensis O. Ktze.*
Active Ingredients:	Theaflavin, Theaflavin-3-gallate, Theaflavin-3’-gallate, Theaflavin-3,3’-digallate
Batch #	TJtf130311
Date of Report:	3/13/2013

Analysis	Specification	Results
Assay by HPLC		
Theaflavins	70%	72.34%
Appearance	Brown powder	complies

Storage +4 °C, Do not freeze.

Shelf life 2 years when properly stored.

13 Dexter Rd., East Brunswick, NJ 08816, http://www.qualityphytochemicalsllc.com;

Phone : (908)510-9277; Fax: (732)390-0524

Email: sales@qualityphytochemicalsllc.com

Supplementary Figure 1

The black tea extract was assayed by routine HPLC methods. It showed the specification of theaflavins as 70% and analyzed the active ingredients of the black tea extract: theaflavins, theaflavin-3-gallate, theaflavin-3’-gallate, and theaflavin-3,3’-digallate.

Confidential. For research only NOT for regulatory filing

LCMS REPORT

Compound ID   :
Sample ID   : TY00073-CHS
Injection Date   : 22. Feb. 2013
Inj. Vol.   : 1.0   ul
Location   : P1-F-01
Acq Method   : D:\DATA\20130221\10-80AB_R.M
Data Filename   : D:\COPY\JJF\CHS\CHS-10-80.D
Instrument&Column: LCMS-Y(11#603)

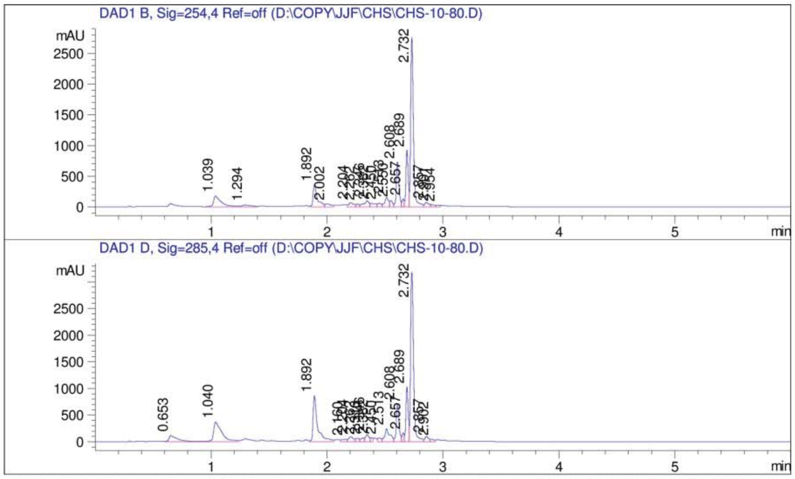

Integration Result

Signal 1 : DAD1 B, Sig=254,4 Ref=off

Peak #	RT [min]	Area	Height	Height %	Width [min]	Area %
1	1.039	875.394	177.280	3.020	0.067	7.857
2	1.294	194.704	31.508	0.537	0.082	1.747
3	1.892	935.042	354.890	6.045	0.038	8.392
4	2.002	178.604	50.116	0.854	0.052	1.603
5	2.204	209.744	68.257	1.163	0.043	1.882
6	2.262	95.253	47.478	0.809	0.027	0.855
7	2.346	318.438	101.441	1.728	0.043	2.858
8	2.382	187.404	58.942	1.004	0.041	1.682
9	2.450	130.281	61.964	1.055	0.030	1.169
10	2.513	378.577	158.294	2.696	0.033	3.398
11	2.550	165.277	99.051	1.687	0.023	1.483
12	2.608	1163.674	722.832	12.312	0.025	10.444
13	2.657	159.675	126.129	2.148	0.019	1.433
14	2.689	1269.828	915.487	15.594	0.022	11.397

Confidential. For research only NOT for regulatory filing

Peak #	RT [min]	Area	Height	Height %	Width [min]	Area %
15	2.732	4549.222	2755.484	46.935	0.025	40.829
16	2.857	186.493	75.057	1.278	0.035	1.674
17	2.901	79.271	37.570	0.640	0.030	0.711
18	2.954	65.257	29.104	0.496	0.032	0.586

Operator:____________

Date:________________

Based on HPLC, theaflavins were calculated as following:

<table>
<tr><td>Compound Name</td><td>TR</td><td>Content</td></tr>
<tr><td>theaflavins</td><td>2.608</td><td>0.12</td></tr>
<tr><td>theaflavin-3-gallate</td><td>2.689</td><td>0.13</td></tr>
<tr><td>theaflavin-3’-galate</td><td rowspan="2">2.732</td><td rowspan="2">0.46</td></tr>
<tr><td>theaflavin-3,3’-digallate</td></tr>
</table>

Supplementary Table 1. Quantitative analysis of black tea extract.

Title: Black tea protects against hypertension-associated endothelial dysfunction through alleviation of endoplasmic reticulum stress

Authors: Wai San Cheang, Ching Yuen Ngai, Ye Yen Tam, Xiao Yu Tian, Wing Tak Wong, Yang Zhang, Chi Wai Lau, Zhenyu Chen, Zhao-Xiang Bian, Yu Huang, Fung Ping Leung

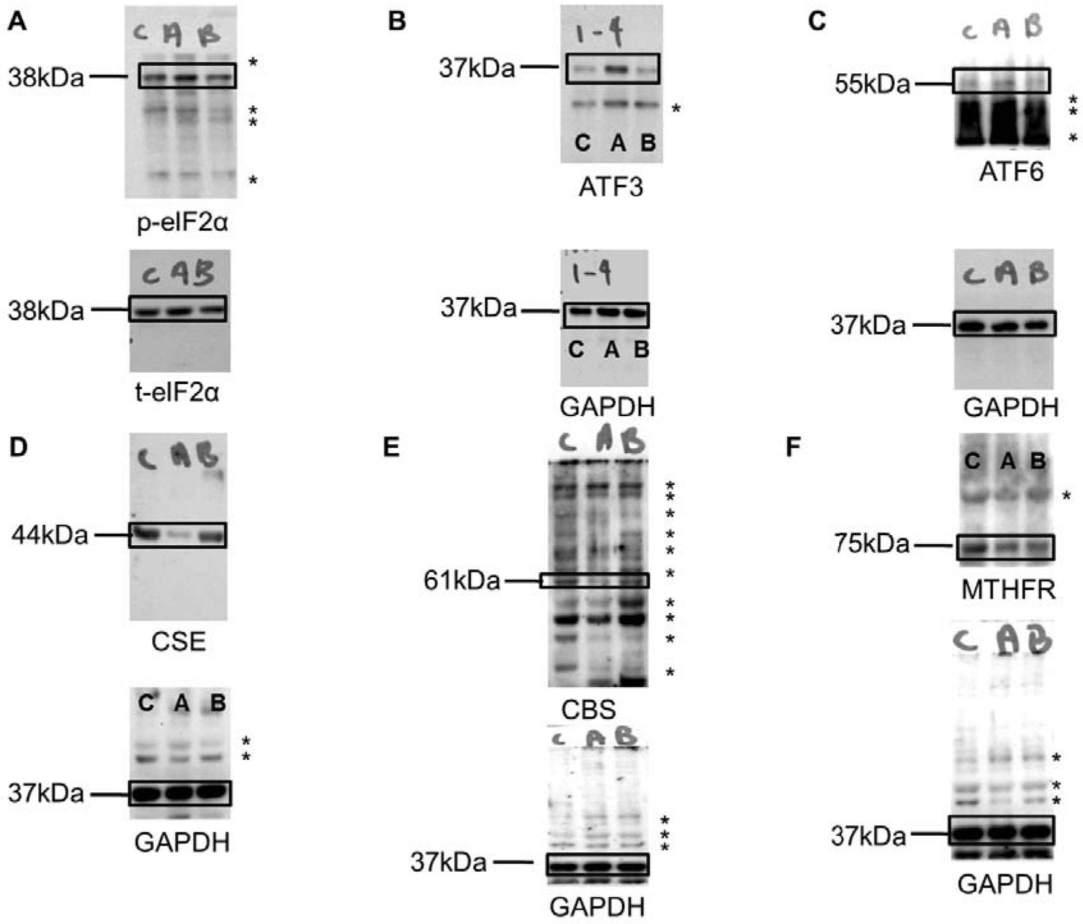

Supplementary Figure 2. Full-length images of Western blots scanned. Rectangular boxes indicate cropped regions used in the representative images. Full-length images of (A) p-eIF2α and t-eIF2α of Figure 4A, (B) ATF3 and GAPDH of Figure 4B, (C) ATF6 and GAPDH of Figure 4C, (D) CSE and GAPDH of Figure 4D, (E) CBS and GAPDH of Figure 4E, (F) MTHFR and GAPDH of Figure 4F. C, control; A, Ang II; B, Ang II+BT. * Nonspecific bands.
